# Neuroprotective Effect of Optimized Yinxieling Formula in 6-OHDA-Induced Chronic Model of Parkinson's Disease through the Inflammation Pathway

**DOI:** 10.1155/2019/2529641

**Published:** 2019-12-21

**Authors:** Renrong Wei, Cuiping Rong, Qingfeng Xie, Shouhai Wu, Yuchao Feng, Ruihua Wang, Zhenhui Dai, Tongxiang Lin

**Affiliations:** ^1^Guangzhou University of Chinese Medicine, Second Clinical Medical College, 232 Waihuan Road East, Guangzhou, Guangdong 510006, China; ^2^Guangdong Provincial Academy of Chinese Medical Sciences & Guangdong Provincial Hospital of Chinese Medicine, Center for Regenerative and Translational Medicine, 111 Dade Road, Guangzhou, Guangdong 510120, China; ^3^Fujian Agriculture and Forestry University, College of Animal Sciences, 15 Shangxiadian Road., Fuzhou, Fujian 350002, China

## Abstract

Parkinson's disease (PD) is characterized by progressive degeneration of dopaminergic neurons in the substantia nigra (SN)-striatum circuit, which is associated with glial activation and consequent chronic neuroinflammation. Optimized Yinxieling Formula (OYF) is a Chinese medicine that exerts therapeutical effect and antiinflammation property on psoriasis. Our previous study has proven that pretreatment with OYF could regulate glia-mediated inflammation in an acute mouse model of PD induced by 1-methyl-4-phenyl-1,2,3,6-tetrahydropyridine. Given that PD is a chronic degeneration disorder, this study applied another PD animal model induced by striatal injection of 6-hydroxydopamine (6-OHDA) to mimic the progressive damage of the SN-striatum dopamine system in rats. The OYF was administrated in the manner of pretreatment plus treatment. The effects of the OYF on motor behaviors were assessed with the apomorphine-induced rotation test and adjusting steps test. To confirm the effect of OYF on dopaminergic neurons and glia activation in this model, we analyzed the expression of tyrosine hydroxylase (TH) and glia markers, ionized calcium-binding adapter molecule 1 (Iba-1), and glial fibrillary acidic protein (GFAP) in the SN region of the rat PD model. Inflammation-associated factors, including tumor necrosis factor-*α* (TNF-*α*), interleukin-1*β* (IL-1*β*), IL-6, inducible nitric oxide synthase (iNOS), and cyclooxygenase-2 (COX-2), were further evaluated in this model and in interferon-*γ*- (INF-*γ*-) induced murine macrophages RAW264.7 cells. The results from the *in vivo* study showed that OYF reversed the motor behavioral dysfunction in 6-OHDA-induced PD rats, upregulated the TH expression, decreased the immunoreactivity of Iba-1 and GFAP, and downregulated the mRNA levels of TNF-*α* and COX-2. The OYF also trended to decrease the mRNA levels of IL-1*β* and iNOS *in vivo*. The results from the *in vitro* study showed that OYF significantly decreased the mRNA levels of TNF-*α*, IL-1*β*, IL-6, iNOS, and COX-2. Therefore, this study suggests that OYF exerts antiinflammatory effects, which might be related to the protection of dopaminergic neurons in 6-OHDA-induced chronic neurotoxicity.

## 1. Introduction

Parkinson's disease (PD) is a common neurodegenerative disorder and is characterized by dopaminergic neurons damage in the substantia nigra (SN) and dopamine (DA) deficiency in the striatum (receive SN dopaminergic nerve terminals projection), which could result in abnormal motor function [1, 2]. In addition to dopaminergic neurons loss, other pathological features have been identified in PD patients, including glial reaction and inflammation [3]. A recent meta-analysis has demonstrated that PD is accompanied by higher peripheral cytokine levels, such as interleukin-6 (IL-6), IL-1*β*, tumor necrosis factor (TNF), and C-reactive protein [4]. In the brain, many inflammatory molecules were also found to be involved in PD, including IL-1*β*, inducible nitric oxide synthase (iNOS), and cyclooxygenase-2 (COX-2) [5]. The expression of iNOS could lead to the production and release of nitric oxide (NO), which may mediate neurotoxicity and be harmful to dopaminergic neurons [6, 7]. These proinflammatory cytokines or neurotoxic substances could be induced by activation of astrocytes and microglia [8, 9]. Since inflammatory response and glial cells activation are associated with PD process, antiinflammatory therapy might be a promising therapeutic intervention for PD [9].

Optimized Yinxieling Formula (OYF) is a Chinese medicine compound modified from the Yinxieling formula, which has been proven to have therapeutical effect and antiinflammation property on psoriasis [10]. The OYF consists of *Curcuma zedoaria*, *Sarcandra glabra*, dark plum fruit, Rhizoma Smilacis Glabrae, *Lithospermum erythrorhizon*, *Paeonia lactiflora*, and *Glycyrrhiza uralensis* [11]. It has been reported that OYF (also called PSORI-CM01) could improve psoriasis [12, 13]. The underlying mechanisms might be through downregulating the keratinocyte cyclin B2 or nuclear factor-kappa B (NF-*κ*B), thus inhibiting cell proliferation or the release of the inflammatory cytokine and chemokine in the keratinocyte [13, 14]. Taken together, these studies suggest that OYF may have antiinflammation property. Given that neuroinflammation plays a crucial role in PD pathology [5], we previously applied the 1-methyl-4-phenyl-1,2,3,6-tetrahydropyridine- (MPTP-) induced mice model to study the effect of OYF in the PD model and found that OYF attenuated SN dopaminergic neuron damage and exhibited antiinflammatory effects [15]. Though the MPTP-induced model is widely used to mimic the PD hallmarks, such as tyrosine hydroxylase (TH, a key enzyme in the DA biosynthesis) immunoreactive neuron loss, this process is acute and may be insufficient for permanent impairment of the SN-striatum pathway [16]. Since the SN-striatum DA pathway dysfunction is associated with PD pathology, in this study, we further applied striatal injection of the 6-hydroxydopamine- (6-OHDA-) induced rat model of PD, in which the loss of the SN-striatum DA pathway is progressive to evaluate the effects of OYF on motor behavioral alterations in PD. In addition, the underlying mechanisms based on neuroinflammation were investigated through *in vivo* and *in vitro* experiments.

Interferon-*γ-* (IFN-*γ*-) induced murine macrophage RAW264.7 is a widely used inflammatory model. RAW264.7 cell line was used to investigate inflammatory-related mechanisms by other researches [17, 18], including the PD associated study [19]. In our study, we hypothesized that the inflammatory mechanism might be involved in the effects of OYF. Thus, IFN-*γ*-induced RAW264.7 was used as an inflammatory model *in vitro*.

## 2. Materials and Methods

### 2.1. Reagents

6-OHDA, apomorphine (APO), and ascorbic acid were purchased from Sigma Aldrich (St. Louis, MO, USA). INF-*γ* was bought from Sino Biological Inc. (Beijing, China). Fetal bovine serum (FBS) and Roswell Park Memorial Institute (RPMI) 1640 medium were obtained from HyClone (Logan, UT, USA) and Invitrogen (Carlsbad, CA, USA), respectively. Rabbit anti-TH antibody, rabbit anti-Iba1 antibody, and anti-GFAP antibody were obtained from Abcam (Cambridge, UK). Immunohistochemical kit (containing hydrogen peroxide, blocking solution, horseradish peroxidase- (HRP-) conjugated secondary antibody, and 3,3-diaminobenzidine (DAB)) was purchased from UNIV (Shanghai, China). SYBR Green Supermix was obtained from Bio-Rad (USA).

### 2.2. OYF and Preparation of OYF Containing Serum (OYFCS)

The OYF is composed of seven Chinese herbs at a specific ratio (Table 1). OYF used for animal experiments was prepared by the Guangdong Provincial Hospital of Chinese Medicine.

To prepare OYFCS for the *in vitro* experiment, adult male Sprague Dawley rats (250 ± 20 g) were used. The animals were bought from the Beijing Vital River Laboratory Animal Technology Co., Ltd. (Beijing, China). All animal experiments were approved and carried out in accordance with the Institutional Animal Care Guidance of Guangzhou University of Chinese Medicine (approval number: 2018004). The rats were orally administrated with OYF (6.45 g/kg) or saline for 3 days. The blood samples were collected from the abdominal aorta of rats under anesthesia and centrifuged at 3000 r/min for 15 minutes. The serums were separated and incubated at 56°C for 30 minutes. 0.22 *μ*m filters were applied to prepare the serum. The final OYFCS or control serums were stored at −20°C until use.

### 2.3. 6-OHDA-Induced PD Rat Model and OYF Administration

Male Sprague Dawley rats (250 ± 10 g) were purchased from the Beijing Vital River Laboratory Animal Technology Co., Ltd. (Beijing, China) and housed in cages under constant temperature (20–22°C) and a 12/12 h light-dark cycle. Chow and water were available freely. After acclimating to the environment for 7 days, the APO-induced rotation test (the method is described in Section 2.4) was conducted before operation [20], and only rats without rotation were used for stereotaxic injection.

All rats were randomly divided into three groups (*n* = 12 per group): sham group (vehicle injection and intragastric administration with distilled water), 6-OHDA group (6-OHDA injection and intragastric administration with distilled water), and 6-OHDA + OYF group (6-OHDA injection and intragastric administration with 12.9 g/kg OYF). All oral treatments began 1 week before surgery and continued for another 8 weeks after surgery. All rats received treatment once a day.

The stereotaxic injections began 2 h after the seventh dose of OYF oral pretreatment. The rats were anaesthetized with 3% pentobarbital sodium (50 mg/kg, i.p) and placed in a stereotaxic apparatus (RWD Life Science Co., Ltd., Shenzhen, China). 6-OHDA (5 g/L, dissolved in normal saline with 0.02% of ascorbic acid) was injected unilaterally into two sites of the right striatum (coordinates from the bregma: site 1: anteroposterior: 0.0 mm, mediolateral: −3.2 mm, and dorsoventral: −7.0 mm; site 2: anteroposterior: −1.2 mm, mediolateral: −4.0 mm, and dorsoventral: −7.0 mm) with a microsyringe at a rate of 1 *μ*L/min. Each site was injected with 2 *μ*L 6-OHDA. After injection, the microsyringe was kept in the place for 10 min and slowly retracted. The sham group rats were injected with the vehicle (normal saline with 0.02% of ascorbic acid).

### 2.4. Behavioral Tests

#### 2.4.1. APO-Induced Rotation Test

APO-induced rotation test was performed according to the protocol described by Ungersteadt [21] with minor modifications. The APO-induced rotation test was conducted before stereotaxic injection [20] to exclude other potential factors which might influence the results. After 6-OHDA injection, the APO-induced rotation test was performed to evaluate the effects of treatments on week 2, 4, 6, and 8, respectively. APO was dissolved in normal saline containing 0.02% of ascorbic acid. The rats were subcutaneously injected with 0.5 mg/kg APO [22] and allowed to habituate for 5 min. The number of contralateral rotation was recorded during a 30-min test session.

#### 2.4.2. Adjusting Steps Test

Adjusting steps test was performed to assess the motor function of the forelimbs on week 8 after the 6-OHDA lesion. Before the test, the rats were handled by the experimenter several times to accustom for 2 days. During the test, the hind limbs, hind part, and one forelimb of the rat were held by the experimenter above the surface, while another forelimb was allowed to touch the surface. The rat was moved forehand or backhand slowly (5 s for 0.9 m), which resulted in adjusting stepping. The number of the steps was recorded for both forelimbs. For each forelimb, the test was repeated twice each day and lasted for 3 days consecutively. The average number of steps in the forehand and backhand directions was calculated, respectively.

### 2.5. Brain Tissue Preparation

After behavioral tests, the rats were anaesthetized with 3% pentobarbital sodium (50 mg/kg, i.p) and perfused transcardially with chilled normal saline. The brains were isolated rapidly and stored at −80°C until real-time PCR experiment was performed. For the immunohistochemistry experiment, the rats were perfused with 4% paraformaldehyde (PFA) after normal saline perfusion. The brains were isolated and fixed in 4% PFA.

### 2.6. Cell Culture and Treatment

Murine macrophages RAW264.7 cells were cultured in RPMI 1640 medium supplemented with 10% FBS and 100 U/ml penicillin-streptomycin in an atmosphere under the condition of 37°C and 5% CO2. RAW264.7 cells were cultured with different doses of OYFCS (2.5, 5, and 10%) or vehicle for 2 h, followed by IFN-*γ* (50 U/mL) for 24 h [23]. The control group cells were exposed to the vehicle alone. Then, the cells were collected for real-time PCR experiment.

### 2.7. Immunohistochemistry

The brain tissues were dehydrated, embedded with paraffin, and cut into 5 *μ*m-thick coronal sections with a microtome (Leica, Germany). The sections were dewaxed, hydrated, and then heated with the citric acid buffer (pH 6.0) for antigen retrieval. 3% hydrogen peroxide was utilized to inactivate endogenous peroxidase for 15 min. The sections were washed thrice in the phosphate buffered saline (PBS) and incubated with a blocking solution for 5 min at room temperature. Primary antibodies (TH 1 : 1000, Iba-1 1 : 2000, and GFAP 1 : 1000) were added onto the sections and incubated for 20 minutes at 37°C. The sections were then washed with the PBS solution three times (5 min each time) and incubated with a primary antibody enhancer for 10 min, followed by another three washes with PBS. HRP-conjugated secondary antibodies were added for a 10-minute reaction at room temperature. The sections were washed with PBS three times before combined with DAB for 5 min. Finally, the sections were washed with water and dehydrated by gradient ethanol. Images were captured by using an Olympus BX61 microscope. Four sections of the midbrain were chosen randomly, with a total of 12 sections per rat. The number of positive-staining cells (for TH and Iba-1) or the positive staining area (for GFAP) in the SN region was analyzed by using the ImageJ software. For TH, the ratio of TH-positive cells in the ipsilateral to in the contralateral SN was calculated [22] and then was normalized to that of the sham group. For GFAP, the percentage of positive-staining area to the image area was calculated.

### 2.8. Total RNA Extraction and Real-Time PCR

Total RNA was extracted from the ipsilateral SN of the midbrain tissue or from RAW264.7 cells with the TRIZOL reagent (Invitrogen, USA) according to the manufacturer's instructions. GoScript Reverse Transcription System (Promega, USA) was used for cDNA synthesis. From this reaction, 5 *μ*l was used as a template for further real-time PCR reaction with iTaq Universal SYBR Green Supermix (Bio-Rad, USA) according to the manufacturer's instruction. All primers used in real-time PCR reactions are listed in Tables 2 and 3. The real-time PCR reactions were performed with the real-time PCR system (Bio-Rad, CFX96, USA). A triplicate analysis was employed for each sample. The real-time PCR system was used to analyze the results (*β*-actin was used as the internal control).

### 2.9. Statistical Analysis

The SPSS 21 software was used for the statistical analysis. All values are presented as the mean ± standard deviation (SD). Differences were analyzed using one-way analysis of variance (ANOVA), followed by least significant difference (LSD) test or Games–Howell test as post hoc testing. A *P* value of less than 0.05 was considered statistically significant.

## 3. Results

### 3.1. Effect of OYF on APO-Induced Rotation in 6-OHDA-Induced PD Rats

**Figure 1:**
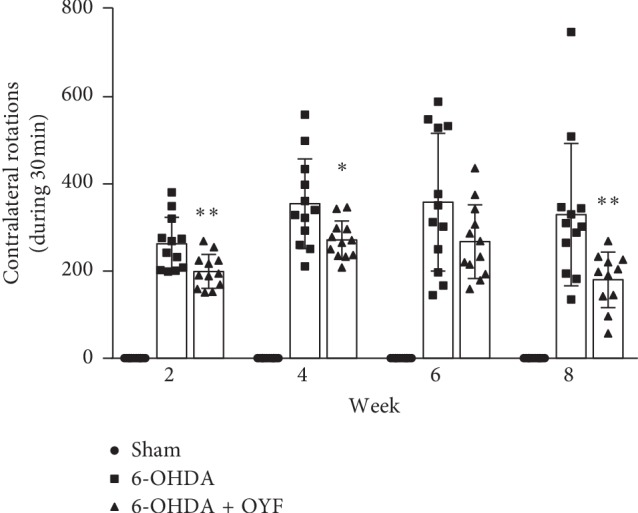
Effect of OYF on APO-induced rotational behavior in 6-OHDA-induced PD rats. The number of contralateral rotations was counted on week 2, 4, 6, and 8 after 6-OHDA injection into the right striatum. Values are expressed as mean ± SD. *n* = 12 per group. ^*∗*^*P* < 0.05 and ^*∗∗*^*P* < 0.01 vs. the 6-OHDA group.

Before stereotaxic injection, none of the rats showed lateral rotation in the APO-induced rotation test. Thus, none of the rats was excluded. After 6-OHDA injection into the striatum, the hypersensitivity of the lesioned striatum was assessed by APO-induced rotation on week 2, 4, 6, and 8. As shown in Figure 1, no rotation was observed in the sham group rats and the total contralateral rotations in the 6-OHDA group were increased. The number of contralateral rotations in the OYF treatment group was significantly reduced on week 2 (*P* < 0.01), week 4 (*P* < 0.05), and week 8 (*P* < 0.01) as compared to the 6-OHDA group. OYF also attenuated the contralateral rotations on week 6 although without significant difference.

### 3.2. Effect of OYF on Motor Function of Forelimbs in 6-OHDA-Induced PD Rats

**Figure 2:**
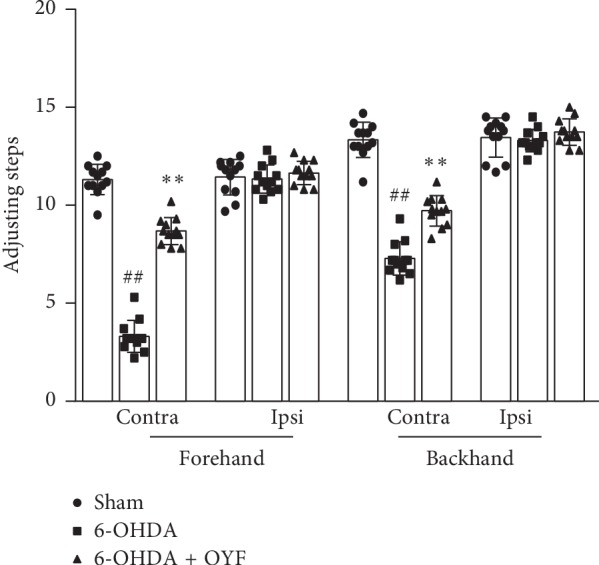
Effect of OYF on motor function of the forelimbs in 6-OHDA-induced PD rats. The number of adjusting steps with the contralateral (contra) and ipsilateral (ipsi) forelimbs in the forehand direction and the backhand direction on week 8 after 6-OHDA injection into the right striatum. Values are expressed as mean ± SD. *n* = 12 per group. ^##^*P* < 0.01 vs. the sham group, and ^*∗∗*^*P* < 0.01 vs. the 6-OHDA group.

On week 8 after 6-OHDA lesion into the right striatum, the 6-OHDA group showed a significant reduction in adjusting steps with the contralateral forelimb in forehand direction (*P* < 0.01) and backhand direction (*P* < 0.01) compared with the sham group (Figure 2). OYF treatment significantly increased the number of adjusting steps with the contralateral forelimb when compared to the 6-OHDA group (Figure 2, (*P* < 0.01)). In contract, no significant difference between groups was observed with the ipsilateral forelimb in both directions (Figure 2).

### 3.3. Effect of OYF on DA Neurons in 6-OHDA-Induced PD Rats

**Figure 3:**
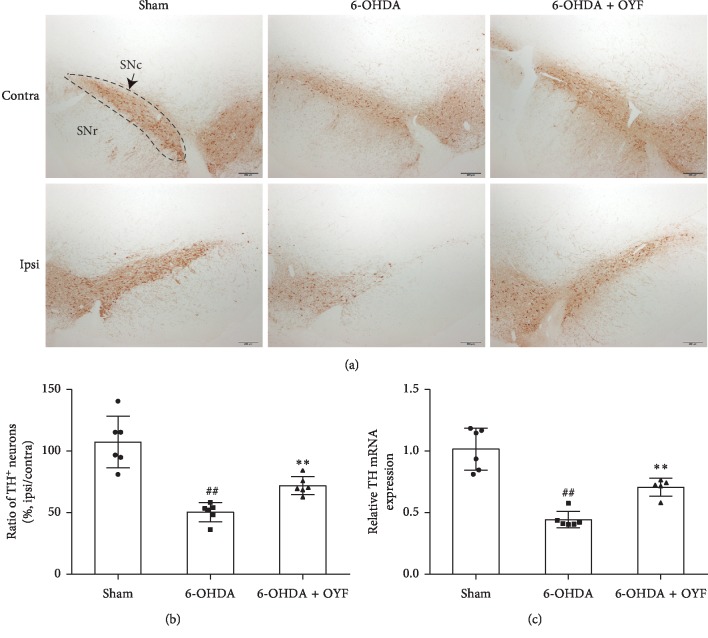
Effect of OYF on TH expression in the SN of 6-OHDA-induced PD rats. (a) Representative images of TH-positive cells in the ipsilateral SN, scale bars = 200 *μ*m. (b) The ratio of TH-positive cells in the ipsilateral (ipsi) to in the contralateral (contra) SN. (c) Relative TH mRNA expression in the ipsilateral SN measured by real-time PCR. Values are expressed as mean ± SD. *n* = 6 per group. ^##^*P* < 0.01 vs. the sham group, and ^*∗∗*^*P* < 0.01 vs. the 6-OHDA group. SNc, SN compacta; SNr, SN reticulate.

Given that the striatum receives DA neuron input from the SN region and that TH is a marker of DA neuron, we detected TH protein and mRNA levels in SN with immunohistochemical staining and real-time PCR. As shown in Figures 3(a) and 3(b), the ratio of TH-positive cells in the SN (ipsilateral to contralateral) was significantly decreased in the 6-OHDA group (50.4 ± 7.7%) compared to the sham group (*P* < 0.01). OYF administration increased the ratio by about 21% compared to the 6-OHDA group (*P* < 0.01). Similar to the results of immunohistochemical staining, the TH mRNA level in the 6-OHDA group was downregulated to about 44% compared to the sham group (Figure 3(c), *P* < 0.01). OYF significantly upregulated the TH mRNA level compared to the 6-OHDA group (Figure 3(c), *P* < 0.01).

### 3.4. Effects of OYF on Glial Cell Activation in 6-OHDA-Induced PD Rats

**Figure 4:**
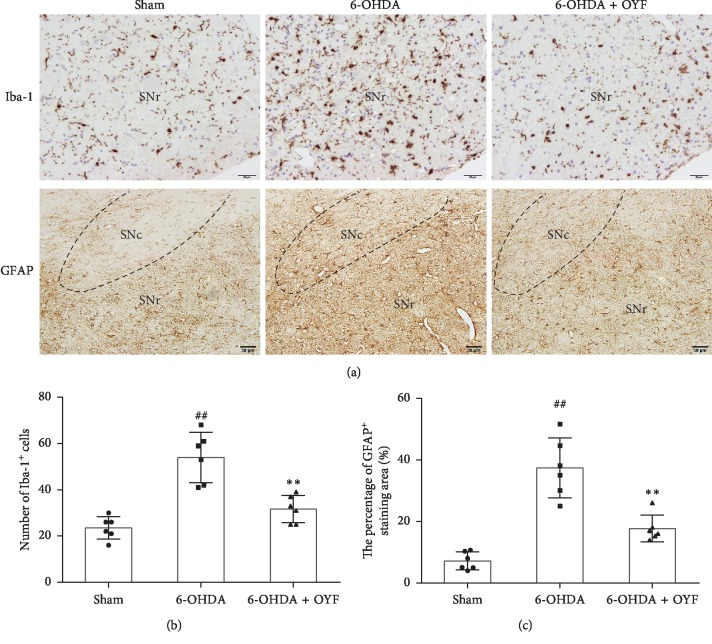
Effects of OYF on Iba-1 and GFAP expressions in the SN of 6-OHDA-induced PD rats. (a) Representative images of Iba-1 and GFAP-positive cells in the ipsilateral SN, scale bars = 20 *μ*m (Iba-1) and 50 *μ*m (GFAP). (b) The number of Iba-1-positive cells in the ipsilateral SN. (c) The percentage of GFAP-positive staining area in the ipsilateral SN. Values are expressed as mean ± SD. *n* = 6 per group. ^##^*P* < 0.01 vs. the sham group, and ^*∗∗*^*P* < 0.01 vs. the 6-OHDA group. SNc, SN compacta; SNr, SN reticulate.

Ionized calcium-binding adapter molecule 1 (Iba-1) and glial fibrillary acidic protein (GFAP) are the markers of microglia and astrocytes, respectively. The number of Iba-1-positive cells in the ipsilateral SN of 6-OHDA group was significantly higher than that of the sham group (Figure 4(b), *P* < 0.01), especially in the SN reticulate subarea (Figure 4(a)). OYF decreased Iba-1-positive cells compared to the 6-OHDA group (Figure 4(b), *P* < 0.01). Similar to the Iba-1, a significantly higher number of GFAP-positive cells was detected in the ipsilateral SN of the 6-OHDA group compared with the sham group (Figures 4(a) and 4(c), *P* < 0.01). Conversely, OYF treatment led to a reduction in GFAP-positive cells as compared to the 6-OHDA group (Figure 4(c), *P* < 0.01).

### 3.5. Effect of OYF on TNF-*α*, IL-1*β*, and IL-6 Expressions in 6-OHDA-Induced PD Rats and in IFN-*γ*-Induced RAW264.7 Cells

**Figure 5:**
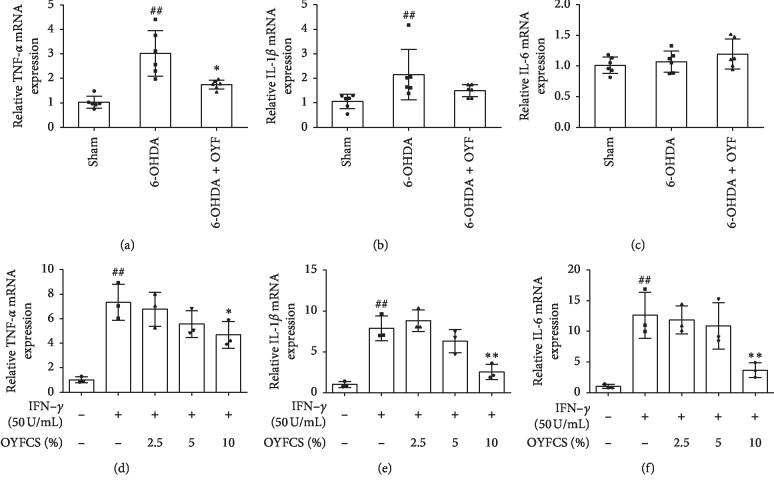
Effects of OYF on TNF-*α*, IL-1*β*, and IL-6 mRNA levels in *in vivo* and *in vitro*. Relative mRNA expression of TNF-*α* (a), IL-1*β* (b), and IL-6 (c) in the ipsilateral SN of rats. Relative mRNA expression of TNF-*α* (d), IL-1*β* (e), and IL-6 (f) in IFN-*γ*-induced RAW264.7 cells. Values are expressed as mean ± SD. *n* = 5-6 per group in the *in vivo* experiment, *n* = 3 per group in the *in vitro* experiment. ^##^*P* < 0.01 vs. the sham group or the control group, and ^*∗*^*P* < 0.05 and ^*∗∗*^*P* < 0.01 vs. the 6-OHDA group or the IFN-*γ* group.

In the *in v*ivo experiment, the mRNA levels of TNF-*α* and IL-1*β* in the lesioned SN of the 6-OHDA group were significantly upregulated when compared to the sham group (Figures 5(a) and 5(b), both *P* < 0.01). OYF significantly attenuated the TNF-*α* mRNA level compared with the 6-OHDA group (Figure 5(a), *P* < 0.05), without a significant decrease in the IL-1*β* mRNA level (Figure 5(b), *P* > 0.05). However, IL-1*β* mRNA in the OYF group had a decrease trend compared to the 6-OHDA group. For the IL-6 mRNA level, no significant difference was observed among groups (Figure 5(c)).

In the *in vitro* experiment, IFN-*γ* significantly increased the TNF-*α*, IL-1*β*, and IL-6 mRNA levels in RAW264.7 cells (Figures 5(d)–5(f), all *P* < 0.01). OYFCS at the dose of 10% significantly downregulated the TNF-*α*, IL-1*β*, and IL-6 mRNA levels (Figures 5(d)–5(f); *P* < 0.05, *P* < 0.01, and *P* < 0.01, respectively).

### 3.6. Effects of OYF on iNOS and COX-2 Expressions in 6-OHDA-Induced PD Rats and in IFN-*γ*-Induced RAW264.7 Cells

**Figure 6:**
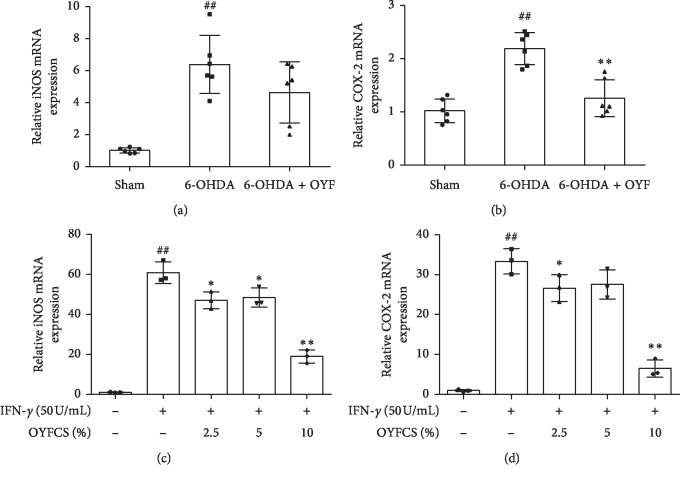
Effects of OYF on iNOS and COX-2 expression in *in vivo* and *in vitro*. Relative mRNA levels of iNOS (a) and COX-2 (b) in the ipsilateral SN of rats. Relative mRNA levels of iNOS (c) and COX-2 (d) in IFN-*γ*-induced RAW264.7 cells. Values are expressed as mean ± SD. *n* = 5-6 per group in the *in vivo* experiment, and *n* = 3 per group in the *in vitro* experiment. ^##^*P* < 0.01 vs. the sham group or the control group, and ^*∗*^*P* < 0.05 and ^*∗∗*^*P* < 0.01 vs. the 6-OHDA group or the IFN-*γ* group.

In the *in vivo* experiment, the mRNA levels of iNOS and COX-2 in the lesioned SN of the 6-OHDA group were significantly elevated compared to the sham group (Figures 6(a) and 6(b), both *P* < 0.01). Though the difference between the OYF group and the 6-OHDA group was not significant, iNOS in the OYF group showed a reduction trend compared to the 6-OHDA group (Figure 6(a), *P* > 0.05). The OYF treatment downregulated the COX-2 mRNA level compared with the 6-OHDA group (Figure 6(b), *P* < 0.05).

In the *in vitro* experiment, IFN-*γ* significantly increased the iNOS and COX-2 mRNA expressions in RAW264.7 cells compared with the sham group (Figures 6(c) and 6(d), both *P* < 0.01). The iNOS mRNA levels were reversed in all OYFCS treatment groups compared to the IFN-*γ* group (Figure 6(c), *P* < 0.05, *P* < 0.05, and *P* < 0.01 for 2.5%, 5%, and 10% dose of OYFCS, respectively). OYFCS at the doses of 2.5% and 10% significantly reduced the COX-2 mRNA expressions compared to the IFN-*γ* group (Figure 6(d), *P* < 0.05 and *P* < 0.01).

## 4. Discussion

Dopaminergic neuron loss and DA deficiency are key pathological features of PD that lead to motor dysfunctions. Though DA replacement therapy reduces clinical symptoms, it cannot slow the neurodegenerative process. Neuroinflammation and immune dysregulation play important roles in the PD pathology [24, 25]. Hence, inflammatory and immune pathways might be potential therapeutic targets for PD. In the present study, we investigated the effects of OYF on motor behaviors and inflammation-related mechanisms in the 6-OHDA-induced rat model of PD and in IFN-*γ*-induced RAW264.7 cells.

OYF is a Chinese medicine formula that has been used for treating psoriasis and shows therapeutical effect [10]. Previous studies of applying OYF in the treatment of psoriasis also reported an improvement effect [12, 13], which might be via the regulation of keratinocyte cyclin B2, inflammatory cytokine, and chemokine release [13, 14]. Psoriasis is an autoimmune disease accompanied by chronic recurrent inflammatory skin symptoms. A resent genome-wide association study has identified several shared loci (including *HLA-DRB5*, *LRRK2*, and *MAPT*) between PD and 7 autoimmune diseases (including psoriasis, rheumatoid arthritis, and so on), suggesting that there were some common genetic risks between PD and autoimmune diseases [26]. A population-based 5-year follow-up study in patients with psoriasis indicated that psoriasis is associated with an increased risk of parkinsonism [27]. Other population-based cohort studies also showed that psoriasis patients had significantly higher risk of developing PD, suggesting a potential relation between PD and psoriasis [28]. Given that inflammation is the common pathology of PD and psoriasis and that the OYF had antiinflammation property in treating psoriasis, our previous study investigated the effects of OYF in the MPTP-induced PD mouse model and found that OYF attenuated the loss of dopaminergic neurons in SN and alleviated the inflammatory response [15]. In addition to the MPTP-induced model, there are other commonly used animal models of PD that employ toxins, including 6-OHDA, which is similar to DA in structure and can specifically kill dopaminergic neurons and their terminals [16]. However, 6-OHDA cannot cross the blood brain-barrier and need to be directly injected into a specific brain location to generate the PD model [29]. Unilateral injection of 6-OHDA in the striatum not only reduced TH (a dopaminergic neuron marker) immunoreactivity, but also enhanced expressions of GFAP (astrocyte marker), OX-42 (microglia marker), and iNOS in both the striatum and SN [30], suggesting that striatal injections of 6-OHDA could progressively destroy dopaminergic neurons in SN [16]. After unilateral injection of 6-OHDA into the striatum, the number of TH-positive neurons in the SN dropped to 39%, 44%, 34%, and 52% of contralateral values at 2, 4, 8, and 16 weeks, respectively [31]. This study suggests that the loss of TH-positive neurons may remain at a relatively high level at 2 to 8 weeks postlesion. Therefore, we utilized unilateral injection of 6-OHDA into the striatum as the PD model and chose 8 weeks as the end point to further confirm the effects of OYF in PD in our study.

Dopaminergic neuron loss in the SN is an important pathological feature in PD. Our data showed that unilateral injection of 6-OHDA in the striatum led to motor dysfunction (Figures 1 and 2) and reduction in TH expression in SN (Figure 3), which is consistent with other research studies [30]. As reported by Blandini et al., APO-induced rotations were present stably and TH-positive cell loss in the ipsilateral SNc was about 51.8% at the fourth week after intrastriatal injection of 6-OHDA [22]. OYF treatment attenuated the abnormal movements and the loss of TH-positive neurons (Figures 1–3), suggesting OYF might have protective effect on dopaminergic neurons thus improving the motor function. It has been reported that TH-positive neurons could be injured by glia-mediated inflammation [32]. Reactive microglia is a prominent pathological feature of PD since an increase of microglia markers (such as HLA-DR and Iba1) was detected in the SN of PD patients [33, 34]. Furthermore, MPTP-induced PD monkeys also showed reactive microglia years after MPTP exposure, suggesting a chronic neuroinflammation process in PD [35]. In addition, MPTP enhanced GFAP immunoreactivity in the SN of mice [36]. Thus, these studies indicated the activation of glia in PD. In our study, the 6-OHDA lesion increased the immunoreactivity of GFAP and Iba-1 in the SN (Figure 4). 1-week pretreatment plus 8-week treatment with OYF significantly inhibits the expression of GFAP and Iba-1 (Figure 4), indicating that OYF might suppress the glia activation. On the contrary, glia activation may further affect the neuronal activity. For example, activated microglia induced neurotoxic reactive astrocytes and resulted in neuronal death [37]. Inhibition of microglia-induced neurotoxic reactive astrocytes showed neuroprotective effect in PD models [38]. Therefore, in addition to the direct impairment of 6-OHDA, glia activation might play a potential role in the loss of TH-positive neurons.

Activation of glia can result in the production of proinflammatory factors, including TNF-*α*, IL-1*β*, and IL-6, elevated levels of which were found in blood from patients with PD as well as from the animal model of PD [36]. Our data showed that striatal injection of 6-OHDA increased the mRNA levels of TNF-*α* and IL-1*β*, but not IL-6 in the SN (Figure 5). OYF treatment reduced the TNF-*α* mRNA level and trended to decrease IL-1*β* mRNA (Figure 5). Furthermore, in the *in vitro* experiment, IFN-*γ* stimulation upregulated TNF-*α*, IL-1*β*, and IL-6 mRNA levels in RAW264.7 cells, whereas a high dose of OYFCS reversed the elevated levels (Figure 5). These results were similar to our previous study in the MPTP-induced mice model and in LPS-induced BV-2 cells [15]. Taken together, these results suggest that OYF exerts an antiinflammation effect in the PD model.

Proinflammatory cytokines, such as IFN-*γ*, IL-1*β*, and TNF-*α*, might be involved in the induction of iNOS [6]. IL-1*β* increased the transcription of the gene encoding TNF-*α* [39]. Decreasing TNF-*α* level reduced movement symptom in the PD animal model [40]. In addition, IL-1*β* and IFN-*γ* regulated the transcriptional activity of the iNOS gene [41]. iNOS stimulated the degeneration of dopaminergic neurons in the MPTP-induced PD model [42]. On the other hand, iNOS-derived NO regulated COX-2 activity [43], which might be caused by a mechanism of iNOS binded to S-nitrosylated COX-2 [44]. Thus, COX-2 is a potential downstream effecter of iNOS [45]. COX-2 induced microglial activation and triggered the release of inflammatory mediators (such as TNF-*α*, IL-1*β*, and IL-6), resulting in dopaminergic neuron damage in Zn-induced parkinsonism [46]. Though COX-2 is induced in the glia in response to inflammation, in neurons it is induced in response to excitatory synaptic activity [47]. Mice lacking COX-2 showed increase in TH-positive neuron survival in the SN in the MPTP-induced model [48], whereas COX-2 inhibitors enhanced human neuroblastoma cell death induced by MPTP [49]. In 6-OHDA model, COX-2 was induced in two neuronal cell lines, leading to upregulation of IL-1*β* [50]. The iNOS, NO, and COX-2 are important inflammatory mediators involved in PD pathology [7, 51]. To confirm whether the antiinflammation effect of OYF involves iNOS and COX-2, we investigated these inflammatory modulators in this study. The results showed that 6-OHDA lesion increased the mRNA levels of iNOS and COX-2 in the SN (Figure 6), and OYF significantly downregulated the COX-2 level (Figure 5). OYF had a trend to decrease the iNOS mRNA levels in MPTP and 6-OHDA models. Additionally, OYFCS also significantly reduced the elevated levels of iNOS and COX-2 induced by IFN-*γ* in RAW264.7 cells (Figure 6). Thus, iNOS and COX-2 may be involved in the antiinflammation effect of OYF in PD.

## 5. Conclusions

In summary, this study showed that OYF reversed the motor behavioral dysfunction in 6-OHDA-induced rats, which might be partly through attenuating dopaminergic neuron loss, inhibiting activation of astrocytes and microglia, and downregulating inflammatory factors. It suggests that OYF might be a potential antiinflammatory agent for preventing or treating PD. However, further research studies are still needed to better understand the mechanism of OYF in PD.

## Figures and Tables

**Table 1:** OYF components.

Components	Ratio
*Curcuma zedoaria*	3
*Glycyrrhiza uralensis*	2
Dark plum fruit	5
*Lithospermum erythrorhizon*	2
*Paeonia lactiflora*	3
*Sarcandra glabra*	5
Rhizoma Smilacis Glabrae	5

**Table 2:** Primers applied in the real-time PCR for *in vivo* experiment.

Genes		Primers (5′–3′)
TH	Forward	CCTTCCAGTACAAGCACGGT
	Reverse	TGGGTAGCATAGAGGCCCTT

COX-2	Forward	CTCAGCCATGCAGCAAATCC
	Reverse	GGGTGGGCTTCAGCAGTAAT

iNOS	Forward	TAGTCAACTACAAGCCCCACG
	Reverse	AGTCACATGCAGCTTGTCCA

TNF-*α*	Forward	ACCCACACCGTCAGCCGAT
	Reverse	CAGAGCAATGACTCCAAAGTAGACC

IL-1*β*	Forward	AGAGCATCCAGCTTCAAATCTCAC
	Reverse	AGGTGCTTGGGTCCTCATCCT

IL-6	Forward	AGCCACTGCCTTCCCTACTTC
	Reverse	CTGTTGTGGGTGGTATCCTCTGT

*β*-Actin	Forward	AGATCAAGATCATTGCTCCTCCT
	Reverse	ACGCAGCTCAGTAACAGTCC

**Table 3:** Primers applied in the real-time PCR for *in vitro* experiment.

Genes		Primers (5′–3′)
TH	Forward	GTCTCAGAGCAGGATACCAAGC
	Reverse	CTCTCCTCGAATACCACAGCC

COX-2	Forward	TTCAACACACTCTATCACTGGC
	Reverse	AGAAGCGTTTGCGGTACTCAT

iNOS	Forward	GTTCTCAGCCCAACAATACAAGA
	Reverse	GTGGACGGGTCGATGTCAC

TNF-*α*	Forward	CCCTCACACTCAGATCATCTTCT
	Reverse	GCTACGACGTGGGCTACAG

IL-1*β*	Forward	GCAACTGTTCCTGAACTCAACT
	Reverse	ATCTTTTGGGGTCCGTCAACT

IL-6	Forward	TAGTCCTTCCTACCCCAATTTCC
	Reverse	TTGGTCCTTAGCCACTCCTTC

*β*-Actin	Forward	GGCTGTATTCCCCTCCATCG
	Reverse	CCAGTTGGTAACAATGCCATGT

## Data Availability

The data are available from the corresponding author upon request.
